# Temporal similarity perfusion mapping: A standardized and model-free method for detecting perfusion deficits in stroke

**DOI:** 10.1371/journal.pone.0185552

**Published:** 2017-10-03

**Authors:** Sunbin Song, Reinoud P. H. Bokkers, Marie Luby, Matthew A. Edwardson, Tyler Brown, Shreyansh Shah, Robert W. Cox, Ziad S. Saad, Richard C. Reynolds, Daniel R. Glen, Leonardo G. Cohen, Lawrence L. Latour

**Affiliations:** 1 NIH/NINDS, Human Cortical Physiology and Neurorehabilitation Section, Bethesda, Maryland, United States of America; 2 Department of Radiology, Medical Imaging Center, University Medical Center Groningen, University of Groningen, Groningen, The Netherlands; 3 NIH/NINDS, Stroke Branch, Bethesda, Maryland, United States of America; 4 NIH/NIMH, Scientific and Statistical Computing Core, Bethesda, Maryland, United States of America; Henry Ford Health System, UNITED STATES

## Abstract

**Introduction:**

Interpretation of the extent of perfusion deficits in stroke MRI is highly dependent on the method used for analyzing the perfusion-weighted signal intensity time-series after gadolinium injection. In this study, we introduce a new model-free standardized method of temporal similarity perfusion (TSP) mapping for perfusion deficit detection and test its ability and reliability in acute ischemia.

**Materials and methods:**

Forty patients with an ischemic stroke or transient ischemic attack were included. Two blinded readers compared real-time generated interactive maps and automatically generated TSP maps to traditional TTP/MTT maps for presence of perfusion deficits. Lesion volumes were compared for volumetric inter-rater reliability, spatial concordance between perfusion deficits and healthy tissue and contrast-to-noise ratio (CNR).

**Results:**

Perfusion deficits were correctly detected in all patients with acute ischemia. Inter-rater reliability was higher for TSP when compared to TTP/MTT maps and there was a high similarity between the lesion volumes depicted on TSP and TTP/MTT (r(*18*) = 0.73). The Pearson's correlation between lesions calculated on TSP and traditional maps was high (r(*18*) = 0.73, p<0.0003), however the effective CNR was greater for TSP compared to TTP (352.3 vs 283.5, t(19) = 2.6, p<0.03.) and MTT (228.3, t(19) = 2.8, p<0.03).

**Discussion:**

TSP maps provide a reliable and robust model-free method for accurate perfusion deficit detection and improve lesion delineation compared to traditional methods. This simple method is also computationally faster and more easily automated than model-based methods. This method can potentially improve the speed and accuracy in perfusion deficit detection for acute stroke treatment and clinical trial inclusion decision-making.

## Introduction

Magnetic resonance imaging (MRI) perfusion imaging is increasingly being used to diagnose and characterize acute ischemia. It has been shown to detect early ischemic changes and large-artery occlusions and differentiate ischemia from stroke mimics [1–3]. In combination with diffusion-weighted imaging (DWI), MRI perfusion imaging has been proposed as a method to identify penumbra in order to select patients who may benefit from revascularization therapy [4]. The amount of tissue that may be salvaged is evaluated by detecting the amount of irreversible ischemic damage with DWI within the area of decreased cerebral perfusion. Although this area of DWI/PWI mismatch does not precisely correlate with the physiological penumbra, several studies have shown that it provides a good estimate of the amount of tissue at risk for infarction [5–7].

Based on the susceptibility effects of an intravenous injected gadolinium based contrast agent, dynamic susceptibility contrast (DSC) perfusion-weighted imaging (PWI) is presently the most used method for detecting perfusion deficits for MRI [8]. Brain tissue with normal perfusion will show an initial steep drop in signal as the contrast agent flows into the brain tissue followed by signal recovery as the contrast agent is diluted. Compared to healthy tissue, under perfused brain tissue will have a signal intensity time-series that is delayed, dispersed and/or decreased [9]. Typically, perfusion deficits are detected and delineated on various parametric maps that are derived from the DSC-PWI images, such as time-to-peak (TTP) and mean-transit time (MTT) [10–13].

The parametric maps are calculations from the raw arterial and tissue enhancement curves through specific mathematical models [14, 15]. TTP is the most straightforward to calculate by identifying the time at which signal intensity reached its minimum, but can be affected by physiological variability in the parenchymal delivery of the contrast bolus. This variability can be corrected by using deconvolution-based algorithms; however, this approach requires selection of an arterial input function. The approach may be problematic in practice since selecting a major artery may not reflect the response at the microvascular level because of differences in collateral flow [16]. Overall, varying contrast injection rates, bolus dispersion, and varying arterial transit times due to reduced cardiac output and flow limiting lesions in the brain feeding arteries can highly affect resultant parametric maps. Ultimately, varying assumptions within models result in wide variability in penumbral calculations and in the prediction of infarcts [12, 14, 17].

With the goal of developing a model-free method for detecting abnormal cerebral perfusion, we explored a different approach that analyzed temporal similarity between signal intensity time-series across the whole brain in each patient. The rationale behind this analysis method was that signal intensity time-series from healthy brain tissue would show high similarity with signal intensity time-series in all other healthy brain tissue, while signal intensity time-series from perfusion lesions would show low similarity with all the healthy brain tissue. The method is model-free and results in scaled and standardized values, with values of perfusion deficit in every patient normalized in relation to their own healthy brain tissue. The aim of this study was to introduce the concept of temporal similarity perfusion (TSP) mapping and to test the ability and reliability of TSP mapping methods to detect and delineate perfusion deficits in acute ischemia.

## Materials and methods

### Study population

This is a retrospective analysis of 20 patients with acute ischemic stroke and 20 patients with transient ischemic attack (TIA). Patients were retrospectively identified from a database of patients admitted and evaluated by the NIH stroke team at MedStar Washington Hospital Center (WHC) in Washington, DC during a two-year period. Data reported in this manuscript was obtained under NIH OHSR Determination (#13285) of Not Human Subjects Research based on “Research Involving Coded Private Information or Biological Specimens” (OHRP, revised Oct 16, 2008) and Guidance on Engagement of Institutions in Human Subjects Research (Oct 16, 2008); was stripped of identifiers and assigned a code prior to analysis.

A vascular neurologist or fellow clinically evaluated all patients. Criteria for inclusion in this analysis were: 1) Acute ischemic patients with baseline pre-treatment MRI with subsequent treatment with standard IV tPA; 2) TIA patients with clinical diagnosis of imaging negative TIA with symptoms resolving within 24 hours of clinical presentation and no treatment with any acute intervention. An equal number of patients based on left versus right vascular territory were selected for the ischemic stroke group. Patients enrolled into a clinical trial were excluded from both groups.

### Imaging protocol

Patients were imaged with a 3 Tesla (Philips Medical Systems, Cleveland, OH) MRI system equipped with an 8-channel coil and using a standardized protocol as part of a clinical pathway for evaluating patients with suspected stroke. The standard clinical imaging protocol included DWI, T2-FLAIR and DSC perfusion imaging, included in this project, along with time-of-flight magnetic resonance angiography (MRA) of the Circle of Willis, T2*-weighted gradient recalled echo for detection of hemorrhage, microbleeds, and thrombi. The duration of the exam was approximately 13 minutes.

Images (excluding MRA) were acquired with FOV = 240, contiguous but interleaved axial-oblique slices aligned with the anterior-posterior commissures using vendor specific technologist supervised auto-align prescription of center and rotation. Slice position was co-localized across DWI, GRE, T2-FLAIR and DSC. For detection of acute ischemia (stroke), a diffusion tensor sequence was used to generate trace-weighted “isotropic” DWI and apparent diffusion coefficient maps. The relevant parameters are as follows; TR/TE = 4400/62 ms, b = 1000 15-direction with one b = 0 image, NEX = 2, FA = 90, 120x120 acquisition matrix, and 40–3.5mm thick slices. For sub-acute ischemia, hyperintense vessel detection, edema, and infarction, T2-FLAIR were acquired with TR/TE/TI = 9000/120/2600 ms, NEX = 1, FA = 90, 240x205 matrix and 40–3.5mm thick slices. Perfusion weighted DSC images were acquired with an echo-planar T2* gradient echo sequence. A single weight-adjusted intravenous dose of 0.1 mmol/kg of gadobutrol (Gd-BT-DO3A, Gadovist, Bayer Healthcare Pharmaceuticals, Wayne, NJ) was administered at 5ml/sec. The scan parameters were: TR/TE = 1000/24 ms, NEX = 1, FA = 70, 80x80 matrix, 20-7mm thick slices, and 80 dynamics.

The TTP and MTT images were calculated using the vendor’s standard perfusion software (R3.2 Release, Philips Healthcare, Cleveland, OH) on the scanner console. An arterial input function (AIF) was identified by sampling multiple points in arteries proximal to the hypoperfused vascular territory based on visual inspection of the dynamic enhancement curves. The mean AIF was used along with circular deconvolution. Temporal and spatial smoothing was applied using the vendor’s software setting of “weak” smoothing for both.

### Temporal similarity mapping

TSP maps were calculated from the raw DSC-PWI images based on a similarity index (the Pearson product-moment correlation coefficient, Pearson’s R). The signal intensity time-series curve for each brain tissue voxel was compared with the other voxels throughout the brain. This method is based on the premise that healthy brain tissue has a uniform perfusion curve and would show high correlation with a Pearson’s R-value close to 1, while under-perfused brain tissue would have an altered signal intensity after contrast injection and thus poor correlation in comparison to healthy tissue. For this method, TSP maps were constructed both with a real-time user-interface as well as automatically through an iterative segmentation process with automatic delineation of the perfusion lesion. Realignment and all the TSP analyses were performed using AFNI (Analysis of Functional NeuroImages).

#### Real-time user-interface for TSP mapping

**Fig 1:**
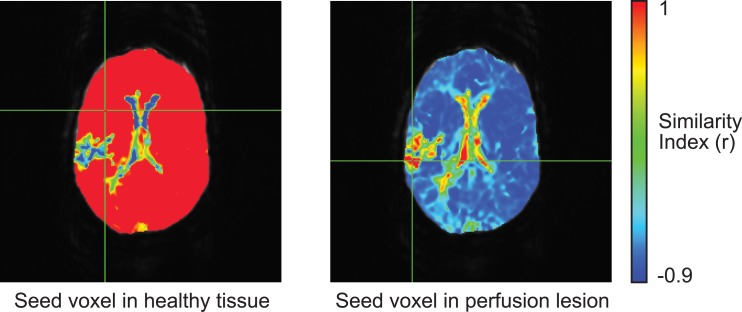
Example of real-time TSP maps in an ischemic stroke patient with a right-sided perfusion deficit. The left panel shows the cursor placed within presumed healthy tissue showing uniform values approaching 1.0 for most of the brain except for focal, heterogeneous perfusion deficit lesions. The right panel shows the seeding voxel within the perfusion deficit. Note that these maps will change in real-time as the investigator moves the cursor around the brain to select a new seeding voxel.

The AFNI (Analysis of Functional NeuroImages) software package was used for real-time TSP mapping by the user (Fig 1A) [18, 19]. This interface generated in real-time (<<1s) a map of Pearson’s correlation between every brain voxel’s perfusion time series and that of a seed voxel. The seed voxel was set interactively through selecting (clicking on) a voxel with the mouse cursor or dragging the cursor over the image for dynamic generation of similarity maps.

#### Automated TSP maps

An unattended, iterative process was used to automatically generate the average signal intensity time-series in all voxels of healthy tissue and a single resulting Pearson’s correlation map. The steps were as follows: 1) DSC perfusion volumes were aligned to the initial volume in order to correct for head motion; 2) a whole brain mask was created that excluded areas of signal loss (calculated from the first pre-contrast perfusion scan) and excluded ventricles (from the isotropic scan); 3) the average signal intensity time-series was calculated for all brain voxels in the whole brain mask; 4) a brain map was calculated as the Pearson’s R correlation at every voxel in the brain with this average signal intensity time-series; 5) a new mask was calculated that only included voxels with Pearson’s R>0.6 and a new average signal time-series was calculated for all voxels in this new mask; 6) steps 4 and 5 were repeated twice with the final similarity map representing the TSP map for every patient. Additional automated TSP maps were calculated at various threshold limits to assess the sensitivity of this threshold (R>0.5, 0.7, 0.8 and 0.9). The entire processing pipeline took <25 seconds per patient on a 64-bit GNU/Linux machine (Intel Xeon CPU E5-2640 6-Core).

### Detection of perfusion deficits

Two experienced stroke image readers (ME, SS) blinded to clinical information read the images. Each reader assessed each TSP, TTP and MTT map by itself in random order to determine the presence of a perfusion deficit. The reader had access to the DWI and FLAIR images. A consensus reading was held for disagreements about the presence of a perfusion deficit. The readers used publicly available software to view the images (MIPAV^™^, NIH, Bethesda, Maryland) and could adjust for window level, contrast, color scheme and magnification of the images. To determine whether a perfusion deficit was present on TSP, the readers evaluated both the automatically generated iterative correlation maps and the real-time generated maps. For the last, the user could freely select and reposition the seed voxel in order determine the presence of a perfusion deficit.

### Quantitative perfusion deficit analysis

Two other expert readers (ML, TB) segmented the perfusion deficits on the MTT and TTP and automatically generated TSP maps with MIPAV^™^. The lesions were semi-automatically segmented on a slice-by-slice basis followed by user-driven editing of the lesion areas. The readers scored each map independently from the other maps. To reduce recall bias, the TSP, MTT and automated TTP images were segmented in separate sessions on different days, each session separated by two weeks. Regions of non-brain tissue, areas of signal loss, and ventricles were identically excluded from all associated TTP, MTT and automatically generated TSP volumes. Automated segmentation was performed using AFNI.

Inter-rater reliability and bias between lesion volumes generated by the two readers (R1 and R2) was compared for automated TSP and TTP and MTT maps. To compare lesion volumes as delineated on TSP maps to those of TTP maps, the lesion segmentations as delineated for each patient by two readers was combined across readers, such that both readers' assessments were considered. We also compared the spatial overlap between the different delineated lesions TSP and TTP maps.

The CNR was determined on TSP and TTP and MTT maps within the healthy tissue and perfusion deficit. Tissue was defined as being within the perfusion deficit if it was included on at least 4 of 6 hand-drawn masks (R1 and R2 on TSP, TTP and MTT maps). This was termed the unbiased perfusion lesion. CNR was defined as: (|Mean signal lesion–Mean signal normal|) / SD signal normal. Effective CNR was calculated as the CNR multiplied by the square root of the number voxels within the unbiased perfusion lesion) with each voxel occupying 0.9375x0.9375x7 mm^3^ of brain volume. Effective CNR values consider that larger lesions require a smaller CNR to be detectable above the Rose criterion.

### Statistical analysis

Paired t-tests (two-tailed) were used to compare TTP and traditional (TTP and MTT) values and were considered significant at p<0.05. Bonferroni correction was applied to correct for multiple comparisons as indicated. Pearson’s correlations were used to relate TTP and TSP values and were considered significant at p<0.05. Mean values are reported as mean +/-SD unless otherwise indicated. Figures plot mean +/-SE unless otherwise indicated.

## Results

Twenty ischemic stroke treated with IV tPA and 20 TIA imaging-negative patients were included. Patients were imaged at a median of 74 minutes from symptom onset (IQR, 41–137 min). The demographics and clinical characteristics are shown in Table 1. The NIHSS score was higher for the patients with an ischemic stroke than with a TIA (NIHSS 15 vs 1, p<0.001). There were no significant differences in age, sex, or time-from-onset (defined by last seen normal) to MR imaging between the ischemic stroke and TIA groups.

**Table 1:** Demographic and clinical characteristics of the study groups.

	All patients (n = 40)	Ischemic Stroke group (n = 20)	Imaging-negative TIA group (n = 20)	Significance (p-value)
Age (years ± SD)	68.4 (±14)	71.6 (±15)	65.2 (±14)	0.140
Sex (n / % female)	27 / 68%	12 / 60%	15 / 75%	0.324
Admit NIHSS [IQR25-75]	5 [1–17]	15 [7–21]	1 [0–1]	<0.001*
Onset (minutes), median [IQR]	74 [41–137]	88 [49–133]	63 [40–148]	0.735
Onset to MRI start time (minutes), median [IQR]	139 [93–205]	150 [93–191]	135 [94–234]	0.776

* significance level of p<0.05

### Detection of perfusion deficits

**Fig 2:**
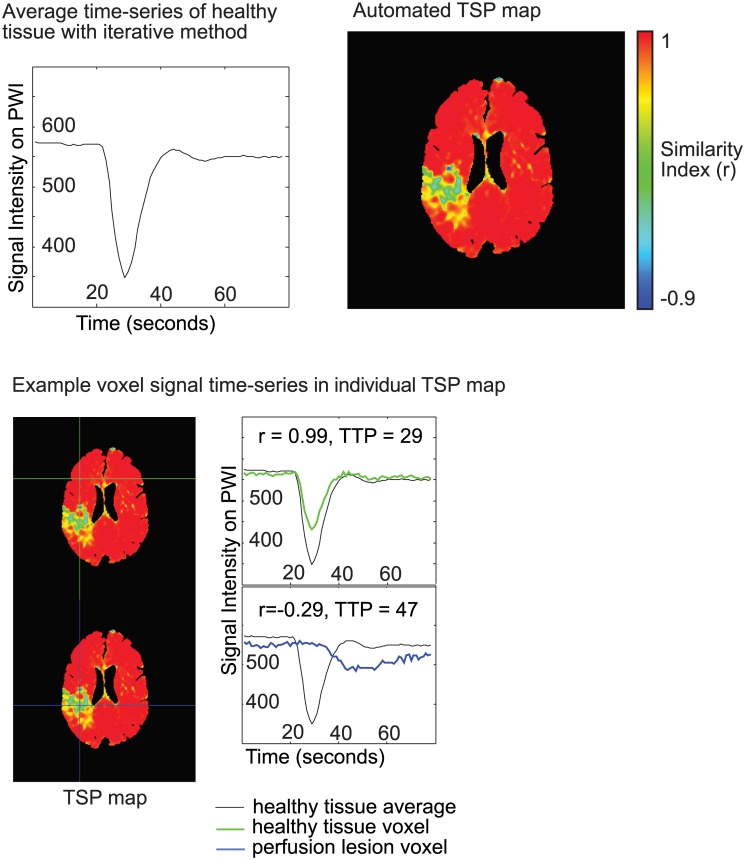
Example of the automated TSP maps in an ischemic stroke patient with a right-sided perfusion deficit. The images were generated using an iterative method that first calculated an average time-series of healthy tissue (top left) to generate a Pearson’s correlation map of all voxels in the brain based on correlation with the average time-series of healthy tissue (top right). Signal intensity time-series for voxels in healthy and under perfused tissue are in the bottom panel. Voxels in healthy tissue demonstrate a signal intensity time-series that is like the average signal intensity time-series for all healthy tissue, while a voxel in the perfusion deficit will have a signal intensity time-series that is delayed, dispersed and/or decreased.

Figs 1 and 2 demonstrate real-time and automated TSP maps in an ischemic stroke patient with a right-sided perfusion deficit. The two expert readers correctly detected all the perfusion deficits in the ischemic stroke patients and were able to differentiate TIA imaging-negative from stroke patients using both real-time TSP mapping and automated TSP maps. No perfusion lesions were detected in the TIA imaging-negative patients. Visually, the real-time TSP maps appeared uniform over most of the brain with values near 1.0 and reduced and negative values within the perfusion deficit (Fig 1, left panel). Real-time TSP maps were robust to seed placement. Seeds placed in healthy tissue showed high correlation with non-lesion areas while seeds placed inside of the perfusion deficit inverted the similarity maps (Fig 1, right panel).

**Fig 3:**
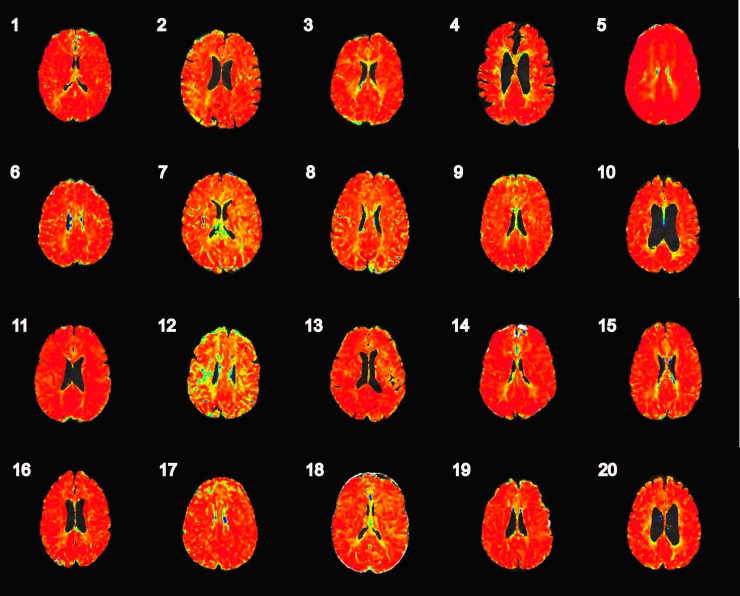
TSP maps in the 20 TIA imaging-negative patients. The TSP maps were uniform with values approaching 1.0 for the whole brain. The color scale for all TSP maps runs from <0 to 1, blue to red.

**Fig 4:**
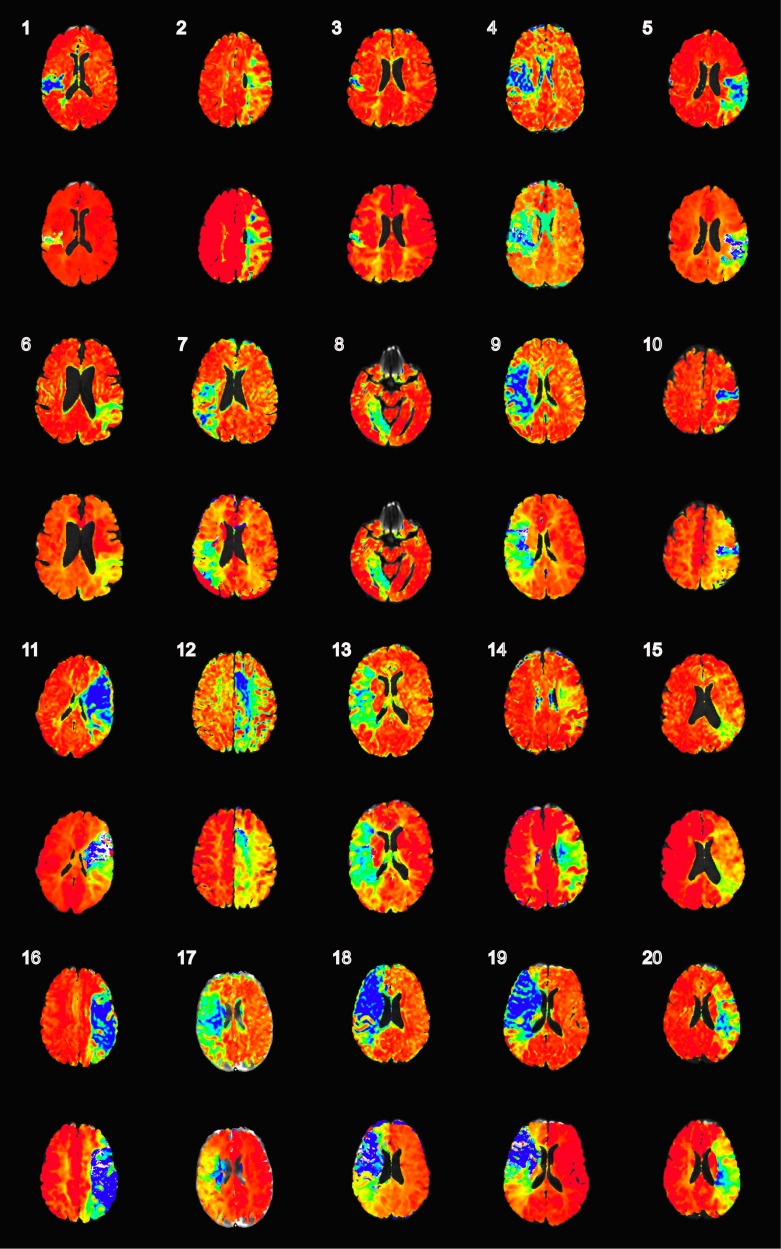
An overview of the automatically generated TSP and TTP maps for all 20 ischemic stroke patients. The slice-of-interest is shown for the mid segment of perfusion deficit. The top row for each patient delineates the TSP map with a Similarity Index (Pearson’s r), color scale from ≤0 to 1 (blue to red). Below the numbered TSP, the corresponding TTP map was individually windowed for each patient. This was done since TTP values are non-standardized in general and can be affected by factors such as speed of bolus injection.

The automated TSP maps were virtually indistinguishable from real-time TSP maps when the seed placement was within presumed healthy tissue (Fig 2). In the TIA imaging-negative patients, automated TSP maps were uniform with values approaching 1.0 over the whole brain (Fig 3). Fig 4 shows the automated TSP for all patients. The images are uniform with values approaching 1.0, except for the area within the perfusion deficit.

### Quantitative perfusion deficit analysis

**Fig 5:**
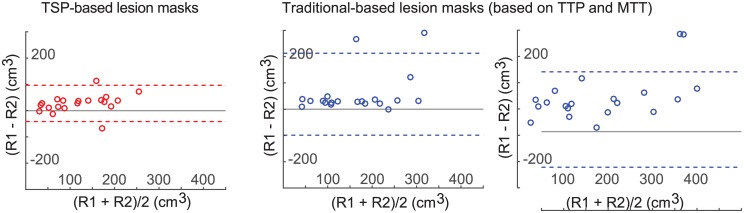
Bland-Altman plots of the inter-rater reliability between the two readers for TSP, TTP and MTT map-based lesion volumes.

Inter-rater reliability for TSP and traditional TTP and MTT map–based lesion volumes are shown with the Bland-Altman plots (Fig 5). The 95% limits of agreement were narrower for TSP (-41.2–96.4 cm^3^) than for TTP (-98.9–213.1 cm^3^) and MTT (-135.7–227.8 cm^3^) map–based lesion volumes.

**Fig 6:**
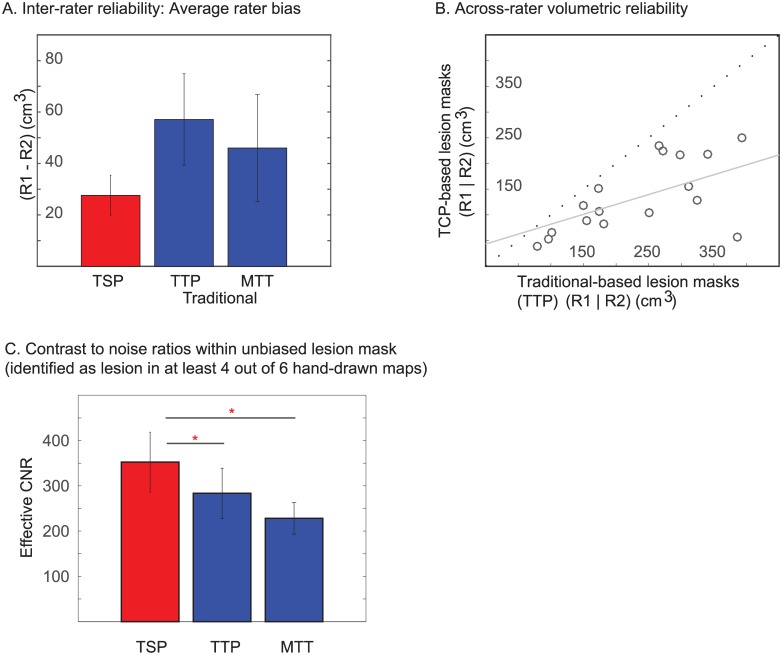
Panel A shows the inter-rater bias measures for TSP, TTP or MTT map-based lesion volumes. Panel B shows that the Pearson's correlation between TSP and TTP map-based lesion volumes was high (r(*18*) = 0.73, p<0.0003). Panel C shows that the effective CNR was greater for TSP (352.3) compared to TTP (p<0.03) and MTT maps (p<0.03).

Inter-rater bias measures for TSP were lower when compared to TTP and MTT map-based lesion volumes, however they did not differ significantly (p>0.4) (Fig 6A). The Pearson's correlation between TSP and TTP-based methods was high (r(*18*) = 0.73, p<0.0003; Fig 6B); however, the effective CNR was greater for TSP (352.3) compared to TTP maps (283.5, t(19) = 2.6, p<0.03.) and MTT maps (228.3, t(19) = 2.8, p<0.03) within the unbiased perfusion deficit (identified in at least 4 of the 6 total drawn lesion masks) (Fig 6C).

**Fig 7:**
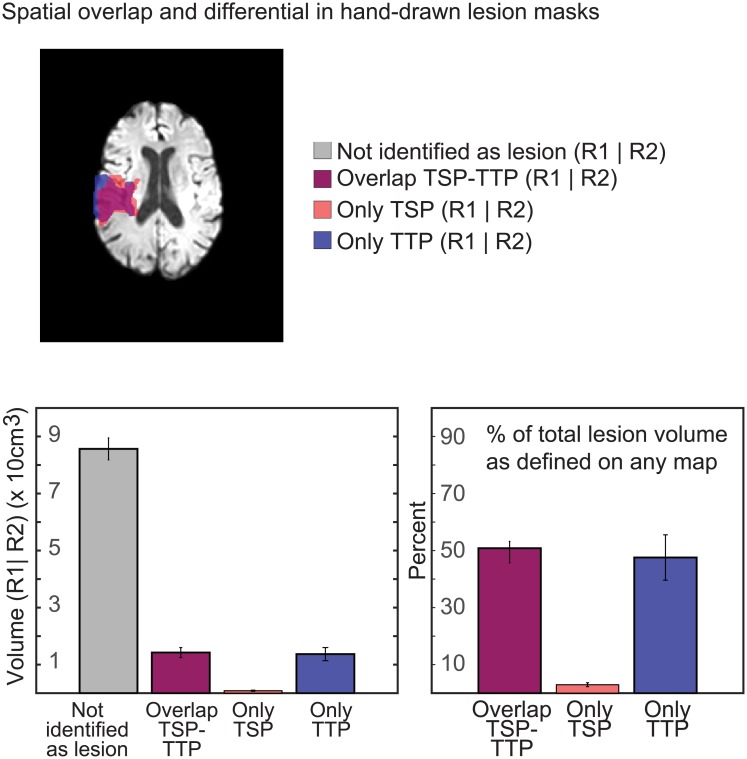
Figure comparing the spatial overlap between lesion volumes drawn on TSP and TTP maps for each patient. Overlap from lesion volumes from each rater was analyzed separately and averaged for each patient.

The average perfusion lesion volume was 150.9+/-77.9 cm^3^ for TSP, 261.4+/-144.2 cm^3^ for MTT and 279.2+/-147.8 cm^3^ for TTP. On average, 142.3±78.4 cm3 of lesion maps were spatially overlapping between the TSP and TTP maps. Of the non-overlapping areas, 8.5±9.0 cm^3^ of the perfusion lesion were only identified on TSP scans and 136.9±102.3 cm^3^ of the perfusion lesions were only identified on TTP maps. Hence, within the total lesion volume defined by either rater on any one of the maps, 50.8±6.8% spatially overlapped on both TSP and the traditional TTP maps while 4.0±5.1% was only defined on TSP-based masks, and 45.2±17.1% was only defined on TTP masks (Fig 7).

**Fig 8:**
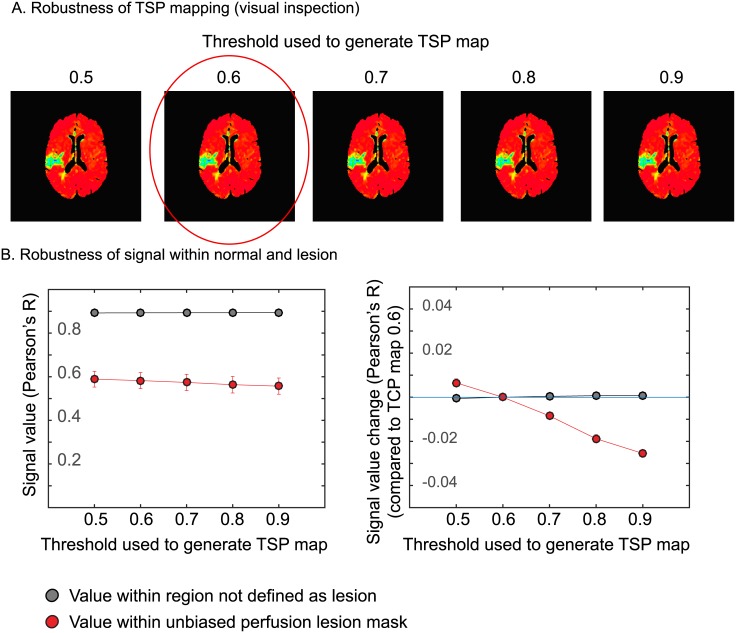
Example of TSP maps with varying Pearson’s R correlation thresholds in a patient with a right-sided perfusion deficit. The red encircled map shows the threshold used for the automated lesion detection. No visual differences were found between TSP maps constructed with these different thresholds (B) Mean signal values for lesion and healthy tissue (based on the unbiased perfusion lesion) showed relatively stable TSP values within TSP maps for healthy and lesion tissue demonstrating robustness. (C) The mean difference in signal between healthy and perfusion tissue increased slightly (by ~0.03 in the 0.9 map compared to the 0.6 map) in TSP values.

Finally, robustness of automated TSP maps was evaluated for sensitivity to different Pearson’s R correlation thresholds during the iterative process. No visual differences were found between TSP maps constructed with these different thresholds (Fig 8). The mean signal values in the perfusion deficit and healthy tissue showed relatively stable TSP values within TSP maps (Fig 8, left panel) demonstrating robustness. However, the mean difference in signal between healthy and perfusion tissue increased slightly (by ~0.03 in the 0.9 map compared to the 0.6 map) if TSP maps were constructed with differing thresholds (Fig 8, right panel).

## Discussion

We compared a new method based on temporal similarity to analyze DSC scans and found TSP mapping was as reliable as TTP mapping for detecting perfusion deficits in acute ischemia. The perfusion lesion volumes segmented in TSP and TTP maps were highly correlated and spatially overlapping. TSP mapping was furthermore found to be more reliable than the traditional TTP method for lesion volume segmentation. This is demonstrated by both increased inter-rater reliability as well as higher CNR ratio for identified lesions. Finally, TSP mapping was found to be robust to different parameters implemented during calculation.

In TTP maps, the absolute values are influenced by factors unrelated to cerebral perfusion, such as speed of contrast agent injection and individual differences in cardiac function and vasculature [14, 16]. The absolute TTP values are therefore highly variable, dependent on the temporal resolution and user driven window leveling is required to detect the perfusion deficit. In contrast, TSP maps are scaled and standardized to the healthy tissue, with a value of approximately 1.0 in all healthy tissue. Tissue with abnormal perfusion will show a lack of correlation and thus a TSP value closer to 0. Hence, in TSP maps, these absolute values are useful metrics that are indicative of healthy versus under-perfused tissue. Deconvolution-based analysis methods also offer scaled and standardized values, such as MTT and CBF, but require model-based parameterizations. Specification of a model requires the selection of an AIF to correct for dispersion effect and nonlinear effects of the tracers in tissue versus bulk blood [20]. The selection of the AIF is however challenging and widely debated [21]. Deconvolution furthermore requires an a priori selection of a deconvolution algorithm to derive a scaled residue function [12]. If the scan duration is very short, and/or bolus arrival is highly delayed, the passage of the bolus may be inadequately sampled, and calculated CBF and MTT values will be artifactually low and noisy [22]. In contrast, TSP analysis is model-free and based on simple metrics of time-series similarity, in this study, Pearson’s correlation coefficients. A priori assumptions or selection of an AIF are therefore unnecessary.

Currently, the accepted standard for detecting perfusion deficits in acute ischemic stroke is by means of deconvolution-based approaches. Other methods, such as (k-means) clustering and cross-correlation analysis have previously been investigated for automated analyses and segmentation purposes. While predominately it is used to analyze DCE perfusion images with patients with renal and pulmonary disease [23–25], Wissmuller et al also presented a method based on neural network clustering that was shown to be able to identify groups of voxels sharing common properties of signal dynamics and delineate perfusion deficits in stroke [26]. Using a similar cross-correlation analysis, Ingrish et al, compared the signal time course of DCE-MRI images in healthy volunteers to automatically segment abnormal lung tissue [27]. In line with these methods, we present an alternative, non-deconvolution based approach and applied it for perfusion deficit detection.

The scaled, standardized, model-free and computationally simple characteristics of TSP mapping allow for automated and rapid lesion segmentation. Clinically, ‘core’ lesions of dead brain tissue are identified on the isotropic DWI scan. The ‘penumbra’ region represents under-perfused but viable tissue that can be potentially salvaged with reperfusion is identified by the PWI/DWI mismatch. TSP and TSP-derived maps along with DWI maps may be employed for automated volumetric and spatial determination of the core versus penumbra in ischemic stroke. Compared to deconvolution, TSP mappings may provide more robust measurements of the perfusion deficits, as it is less dependent on complex post-processing algorithms [12, 14, 17]. With user-driven seed points, the viewer can interactively evaluate the brain tissue and better appreciate subtleties in the data.

This study has limitations. The randomly selected ischemic stroke patients had relatively larger perfusion deficits on average. Therefore, the results cannot be generalized to smaller perfusion deficits, and further research is needed to investigate its use in a generalized population of patients with ischemic stroke. Our study indicates that TSP mapping can be used to detect perfusion deficits in small samples of imaging positive ischemic stroke patients; however, we have not yet investigated whether this method can be used clinically for establishing if a patient subsequently reperfuses after revascularization therapy. Furthermore, in our study we did not investigate whether TSP mapping can differentiate hypoperfused tissue from irreversibly damaged tissue.

## Conclusions

Perfusion deficits can be reliably detected in acute ischemia by analyzing the temporal similarity between signal intensity time-series across the whole brain in perfusion-weighted imaging. Temporal similarity perfusion (TSP) mapping is a model-free standardized method for perfusion deficit detection and can reliably detect and delineate perfusion deficits in acute ischemia. Because of its improved speed and accuracy in perfusion deficit detection, this method can potentially be used for acute stroke treatment decision-making and clinical trial inclusion.
